# Desmoid Fibromatosis in Pediatric Patients: Management Based on a Retrospective Analysis of 59 Patients and a Review of the Literature

**DOI:** 10.1155/2012/475202

**Published:** 2012-08-05

**Authors:** Caroline Oudot, Daniel Orbach, Véronique Minard-Colin, Jean Michon, Pierre Mary, Christophe Glorion, Sylvie Helfre, Jean-Louis Habrand, Odile Oberlin

**Affiliations:** ^1^Pediatric Oncology Department, Hôpital de la mère et de l'enfant, 8 rue Dominique Larrey, 87042 Limoges, France; ^2^Adolescent and Pediatric Oncology Department, Institut Curie, 75005 Paris, France; ^3^Pediatric Department, Institut Gustave Roussy, 94800 Villejuif, France; ^4^Pediatric Surgery Department, Hôpital Trousseau-Assistance Publique, 75012 Paris, France; ^5^Pediatric Surgery Department, Hôpital Necker-Enfants-Malades-Assistance Publique, 75012 Paris, France; ^6^Radiotherapy Department, Institut Curie, 75005 Paris, France; ^7^Radiotherapy Department, Centre Hospitalier Universitaire de Caen, 14000 Caen, France

## Abstract

*Background*. Only limited data are available concerning desmoid tumor in children. *Methods*. Fifty-nine children and adolescents with desmoid tumor treated in 2 French cancer centers with a very long followup were retrospectively reviewed. *Results*. Median age was 6 years (range, 0–15). Tumors mainly involved the limbs (42%). Five cases occurred in a context of genetic disorder. Surgery was first-line treatment in 80% of cases. Resection was microscopically complete in 3 patients (pts), with a microscopic residue in 19 pts and a macroscopic residue in 35 cases. Various adjuvant therapies were used. Overall response to all systemic therapies was 33%. Thirty-eight patients developed one or more recurrences or progressions. After a median followup of 8.5 years, 34 patients were alive in complete remission (CR), including 16 first CR. Seven patients died, 6 from refractory disease and 1 from colorectal carcinoma in a genetic context. Ten-year progression-free survival (PFS) and overall survival were 31% and 88%, respectively. In univariate analysis, age less than 10 years and head-neck site were favorable prognostic factors for PFS. *Conclusions*. When surgery is required, surgical margins must be negative. Low-dose chemotherapy can be proposed as adjuvant therapy. Prospective trials must be developed to evaluate long-term response and side effects.

## 1. Introduction

Desmoid fibromatosis or desmoid tumor (DT), also called deep fibromatosis or aggressive fibromatosis, is a rare disease. The annual incidence is estimated to be 0.2 to 0.4 per 100,000 inhabitants. This benign but monoclonal proliferative soft tissue lesion arises from deep fascia or soft tissues and is derived from mesenchymal stem cells [1, 2]. Two relative incidence peaks are reported in the literature: 6 to 15 years and between puberty and the age of 40 [3]. The etiology is unknown, but DT may be associated with trauma and occurs as a feature of Gardner's syndrome or familial adenomatous polyposis coli (FAP) [4, 5]. These tumors may be multifocal. They have a locally aggressive behavior with no distant metastases, but a tendency for local recurrence after therapy [5]. 

Recurrence of DT is difficult to predict, but seems to be correlated with the quality of surgery and the use of radiation therapy [6–8]. Few data are available in children, but the course of the disease appears to be fairly similar to that observed in adults [3, 7, 9–12]. Depending on the initial with invasion of muscle and fascia, complete surgery is often difficult to achieve, even in children, with less than 25% of microscopically complete resections at diagnosis [3]. Although the standard first-line treatment is still surgery, experience shows that the risk of local relapse remains high even in the presence of clear margins (almost 30% in this case). This finding has led some teams to deliver systematic adjuvant radiation therapy, but recurrences can occur in 19 to 25% of cases even with this therapy. Other nonsurgical approaches have been initiated for unresectable or recurrent tumors: conventional chemotherapy, hormonal therapy, nonsteroidal anti-inflammatory drugs (NSAIDs), radiofrequency ablation, cryoablation, or limb salvage with isolated perfusion [13]. Due to the immature organs of children, the long-term effects of all these therapies, including the risks of cosmetic and functional sequelae after mutilating surgery, must be taken into account in this benign chronic disease [7, 8]. Furthermore, due to the high risk of relapse despite therapy, a wait-and-see attitude has been recently proposed in adults as first-line therapy in order to restrict radical surgical indications to patients with symptomatic disease or documented progression [13, 14]. The respective roles of all these strategies have yet to be clearly defined in adults and in children.

In order to more clearly understand childhood DT, a retrospective study was conducted to describe the characteristics of pediatric patients (pts) with DT with long-term followup, treated in 2 large centers in France. The aim was to evaluate the efficacy of treatments and the outcome of these patients and to propose, after additional analysis of data of the literature, treatment guidelines for children with this rare disease.

## 2. Patients and Methods

### 2.1. Patients and Therapies

 A two-center retrospective study was conducted in all patients under the age of 16 years with histologically confirmed DT, treated in the Institut Gustave Roussy, Villejuif, and the Institut Curie, Paris, from 1976 to 2005. Complete clinical, therapeutic, pathological and radiological records were reviewed in detail for each patient. Initial tumor extent was assessed by ultrasound, and/or computed tomography (CT), and/or magnetic resonance imaging (MRI). No specific treatment guidelines were available and treatment strategies changed to a certain degree over the years. Surgery was performed first when complete resection appeared to be possible. If not, various neoadjuvant therapies, such as radiation therapy or hormonal therapy (antiestrogens), nonsteroidal anti-inflammatory drugs (NSAIDs) or chemotherapy, were delivered [9–11]. Hormonal therapy was not specifically based on the presence of hormonal receptors, as response did not appear to be strictly related to estrogen receptor status [15]. In some patients with a high risk of recurrence, especially after incomplete surgery, adjuvant therapy was delivered. No specific clinical trial was opened for any of the drugs delivered during the study.

### 2.2. Definitions

Complete response (CR) was defined as complete radiological and clinical disappearance of all tumor lesions after surgery, medical treatment or radiation therapy [16]. Partial response (PR) was defined as a decrease of more than 25% of the tumor diameter on radiological and/or clinical assessment. Stable disease (SD) was defined as absence of decrease or an increase of less than 25% of the diameter without the appearance of any new lesions. Progressive disease (PD) was defined as an increase of more than 25% in the lesion and/or the appearance of new lesions. Relapse was defined as recurrence of a tumor in previous CR at least 1 month after the end of treatment. The primary tumor site was classified into the following 4 groups: head and neck, trunk wall, deep trunk, and visceral location or limbs [17]. The status of resection margins in surgically treated patients was reclassified according to the UICC R classification [18]. In order to allow comparisons with other publications, tumors were retrospectively staged according to the Intergroup Rhabdomyosarcoma Study (IRS) postsurgical staging system used for pediatric rhabdomyosarcoma: IRS group I patients are those who underwent complete tumor resection (similar to histologically free margins, for example, more than 3 to 5 mm of clear margins: UICC-R R0), IRS group II patients are those who underwent resection with microscopic residual disease (UICC-R R1), and IRS group III patients are those who underwent resection with macroscopic residual disease (UICC-R R2) or just biopsy [19]. 

### 2.3. Statistical Analysis

Overall survival (OS) was defined as the time from the start of treatment to the time of last followup or death. Progression-free survival (PFS) was defined as the time from the start of treatment to disease progression or relapse. OS and PFS curves were calculated using the Kaplan-Meier method [20]. Log-rank test was used to identify the prognostic significance of clinical factors. A proportional hazards ratio was calculated by a Cox proportional hazards model adjusted for significant prognostic variables (*P* < 0.05) in the univariate model for the overall population.

## 3. Results

### 3.1. Clinical Features

Fifty nine children with DT treated between 1976 and 2005 were included in this study. Clinical characteristics are detailed in Table 1. This population presented a male predominance with a sex ratio of 1.56:1. Median age at diagnosis was 6.75 years (range, 0–15). Seven infants had symptoms before 1 year of age. The tumor involved the limbs in 25 patients (42%), including 4 buttock tumors and the head and neck in 17 patients (28%). Deep trunk primaries included intrathoracic or mediastinal (*n* = 2) or intra-abdominal (*n* = 4) sites. No mesenteric tumor was observed in this series. All patients had a solitary lesion, except for 2 patients with multiple sites. Of the 17 patients with a head and neck primary, 16 were under the age of 10 years. The age distribution of patients with a primary in a site other than the head and neck was well balanced: 20 patients were under the age of 10 and 20 were older than 10. Five cases occurred in a context of a genetic disorder: Gardner's syndrome (*n* = 2) or FAP (*n* = 3), including 2 patients with a family history of Gardner's syndrome (*n* = 1) or FAP (*n* = 1). Another patient had a family history of DT with no identified genetic disorder. Four other patients had associated congenital bone abnormalities (2 fingers, 1 toe, and 1 tibia). In two cases, DT occurred on the scar of a previous local surgical procedure. Another patient developed DT at the site of an appendectomy scar.

### 3.2. Initial Treatment

First-line treatment was surgery in 47/59 cases (80%), while simple biopsy was performed in the other 12 children.

#### 3.2.1. Initial Surgical Approach (*n* = 47)

Initial surgery consisted of tumorectomy, but, when known, surgical margins were histologically tumor-free (R0) in only 3 patients and microscopically positive (R1) in 19 pts. Macroscopic residue (R2) was present in 23 pts. Margins were unknown in 2 cases. IRS group staging is shown in Table 1. Surgery was the unique first-line treatment in 35 children (3 R0, 16 R1 and 16 R2).

#### 3.2.2. Adjuvant Therapy (*n* = 12)

Twelve patients received adjuvant therapy after initial surgery: nine patients received local radiation therapy, to an R2 residue in 4 cases, an R1 residue in 3 cases and to unknown margins in 2 cases. Four of these patients maintained a first continuous complete remission (CCR1) and 5 patients relapsed (3 with initial R1 residue, 1 with R2 residue, and 1 with unknown margins). Details on overall radiation therapy according to disease status and timing of RT are indicated in Table 2. Three patients with R2 residue received adjuvant chemotherapy. One patient achieved CR after 2 courses of ifosfamide, dactinomycin, and vincristine (IVA) for a total of 9 months and maintained a CCR1 11 years after the end of therapy, another patient achieved PR after 1 course of vincristine-ifosfamide and remained progression-free for more than 7 years after the end of treatment, and the last patient achieved SD after 3 months of chemotherapy (IVA and vinblastine-methotrexate) but presented PD 2 months after discontinuation, which was treated by another 6 months of vinblastine-methotrexate. After a transient PR, the patient progressed and required second surgery to an R1 residue. He relapsed 14 months later, was treated by R1 surgery with adjuvant radiation therapy, and subsequently remained in persistent CR 7 years later. 

#### 3.2.3. Neoadjuvant Therapy (*n* = 7)

 Neoadjuvant chemotherapy was delivered for 1 to 5 months before surgery to 7 of the 12 patients undergoing initial biopsy. Six of these patients achieved CR with delayed surgery (3 R0 and 3 R1). One received further adjuvant chemotherapy (due to R0 residue) and only one relapsed (R1 residue) 7 months after surgery. The last patient achieved PR with an R2 residue after surgery that remained stable without further therapy 3 months later. Overall details on response are indicated in Table 3.

#### 3.2.4. Other Strategy (*n* = 1)

One IRS III group patient was only treated medically after biopsy (antiestrogens and low-dose chemotherapy) and achieved PR. This patient has never been operated and had a stable tumor after more than 18 months off therapy.

#### 3.2.5. “Wait-and-See” Strategy (*n* = 4)

Four patients were treated by a “watch-and-see” strategy after biopsy. As their tumors failed to decrease in size or increased, three of them received delayed medical treatment after a period of 8 months, 10 months or 4 years, comprising hormonal therapy and/or chemotherapy followed by surgery. All resections were microscopically incomplete (R1 residue). One of these patients also received adjuvant radiation therapy. All 3 patients relapsed, 4 to 37 months after the end of therapy. The last patient was never treated after the biopsy. This patient had multiple sites of DT and presented stable disease 6 years after the diagnosis without therapy.

#### 3.2.6. Outcome after First-Line Therapy

**Figure 1:**
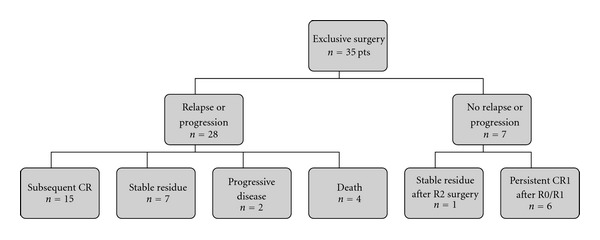
Outcome of the 35 patients initially treated by exclusive surgery. Abbreviations: CR: complete remission; R2: macroscopic residue; R0: no residue; R1: microscopic residue.

**Figure 2:**
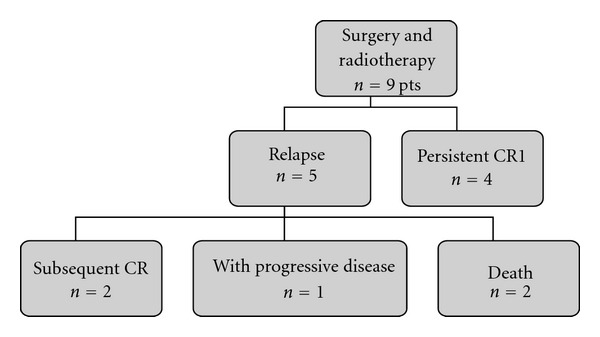
Outcome of the 9 patients initially treated by surgery and adjuvant radiation therapy. Abbreviations: CR: complete remission.

After completion of first-line therapy, 39 patients (66%) were in complete radiological remission and 16 patients (27%) remained in continuous first CR (CCR1). Five patients (9%) had initial PR and presented a stable residue without progression. Thirty-eight patients (64%) developed one or more recurrences or progressions. The median time to first recurrence or progression was 1.13 years (range, 0–6.6 years). Tumor recurrence or progression was treated by second-line or subsequent therapy. The risk of relapse according to the initial IRS status is indicated in Table 1. Fifteen patients received radiation therapy as second-line or subsequent therapy (Table 2). Three of these patients had previously received first-line radiation therapy. Fourteen patients received chemotherapy after the first relapse: as adjuvant therapy after further surgery in 4 cases (1 R1 residue, 2 R2, and 1 unknown margins), or as neoadjuvant therapy before further surgery in 6 cases (1 R0, 3 R1, 1 R2, and 1 unknown margins), or alone in 4 cases. Details concerning the outcome of patients according to first-line therapy are indicated in Table 1 and Figures 1 and 2. Only 6/35 patients (17%) remained in CR1 after exclusive surgery and only 4/9 patients (44%) remained in CR1 after surgery and adjuvant radiation therapy.

#### 3.2.7. Overall Response to Systemic Therapies

Thirty two patients received chemotherapy for macroscopic disease, as first-line therapy for unresectable primary tumor (*n* = 11), after incomplete surgery (*n* = 3), or after relapse or progression (*n* = 18). Various combinations of cytotoxic drugs were used at various times (Table 3). The most commonly used combination was a weekly low-dose combination of vinblastine (6 mg/m^2^) and methotrexate (30 mg/m^2^) given to 15 patients and combined with tamoxifen in another 6 patients. Other combinations included vincristine combined with dactinomycin administered to 9 patients, IVA, or VAC (cyclophosphamide, vincristine, dactinomycin) delivered to 13 and 5 patients, respectively; 5 patients received doxorubicin-based combinations, and 2 received imatinib. Hormonal therapy (tamoxifen) was used 15 times in 9 patients, alone (*n* = 7), in combination with chemotherapy (*n* = 7) or in combination with radiation therapy (*n* = 1). One of these 15 patients achieved CR with tamoxifen alone, 3 patients achieved PR (in combination with chemotherapy in 2 cases and after hormonal therapy in combination with radiation therapy in a case), 6 patients presented SD, and 3 patients presented PD. Response was unknown in 2 cases.

As some patients received more than 1 line of chemotherapy, response was evaluable 64 times. Details of specific response according to the type of regimen are indicated in Table 3. An overall significant response (CR or PR) was documented in 21 pts (33%). Stable disease was the best response in 29 cases (45.5%).

#### 3.2.8. Outcome after Radiation Therapy

Overall, radiation therapy (RT) was delivered 25 times to 21 patients (Table 2). The median dose delivered to the primary site was 50 Gy (range, 36–56 Gy). The outcome of patients after surgery followed by radiation therapy is indicated in Figure 2. Overall, RT was delivered to macroscopic residual disease (R2 margins or neoadjuvant therapy) in 12 patients, resulting in long-term local control in 6 patients (first CCR: 3; subsequent CCR: 3). Among the 12 cases of relapse or progression observed after RT, 60% occurred within the irradiated field. The median time to progression after RT was 12 months (range, 0–45 months). Four patients received delayed second irradiation after disease progression (Figure 2).

#### 3.2.9. Outcome

**Figure 3:**
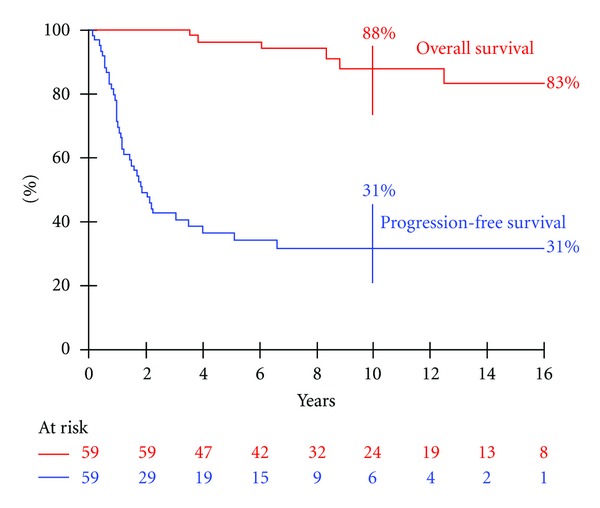
Survival rates in the overall population of children with desmoid tumor (59 patients).

Median followup of the cohort was 8.42 years (range, 2.2–27.9). The 10-year PFS rate of the 59 patients was 31% (95% CI, 20 to 45) and the 10-year OS rate was 88% (95% CI, 74 to 95) (Figure 3). Overall, at the end of followup, 34 patients were alive in CR (16 in 1st CR and 18 in subsequent CR). Fourteen were alive with a residue after a median followup of 60 months (range, 6–130) since last progression and 25 months (range, 0–155) after the last treatment. Four patients were alive with progressive disease. Seven patients had died after a median interval of 8.4 years (range, 3.5–19.1) after the diagnosis. Six patients died from refractory progressive disease despite numerous lines of medical treatment, surgery, and radiation therapy. These patients had mediastinal (*n* = 1), head and neck (*n* = 1), dorsal trunk wall (*n* = 3), and intra-abdominal (*n* = 1) sites. A patient with a head and neck DT remained in CR for 8 years, but died from an associated colorectal carcinoma in a context of FAP. Overall, 3 survivors underwent radical mutilating surgery after relapses despite medical treatment, comprising amputation (finger, *n* = 1; distal part of hand, *n* = 1; thigh, *n* = 1). 

#### 3.2.10. Prognostic Factors

**Figure 4:**
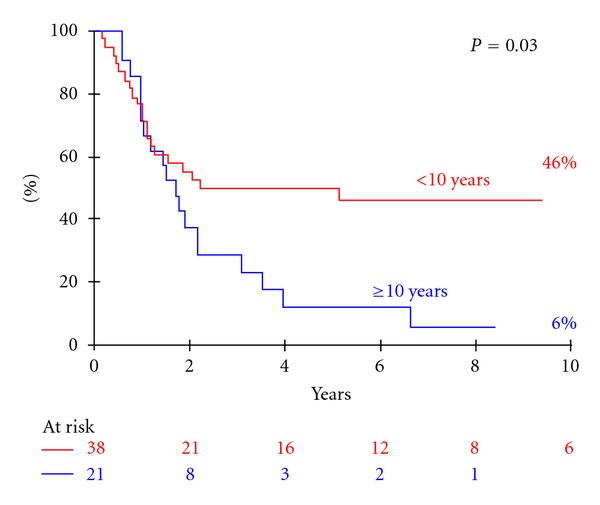
Progression-free survival of the 59 patients according to age (±10 years).

**Figure 5:**
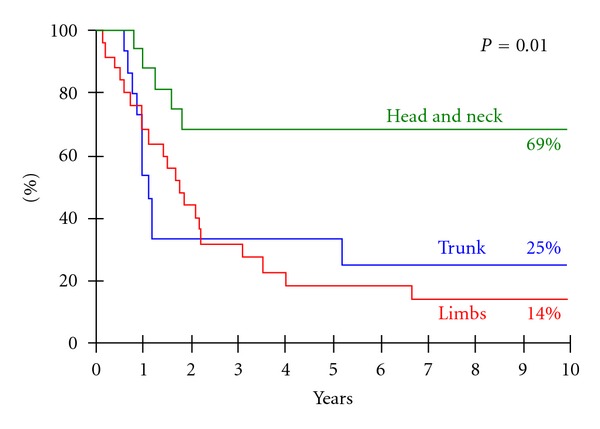
Progression-free survival of patients with desmoid tumor according to initial site.

In univariate analysis, younger age and head and neck sites were favorable prognostic factors for PFS. The 10-year PFS rate was 46% for children younger than 10 versus 6% for older children (*P* = 0.03—Figure 4). The 10-year PFS for patients with a head and neck (H/N) primary DT was better than for those with a limb or trunk DT (69% versus 14% versus 25%, resp., *P* = 0.01—Figure 5). Gender had no impact on PFS. The status of surgical margins did not appear to be a prognostic factor, as IRS staging was not found to be a significant prognostic factor for PFS (5-year PFS: 33% versus 16% versus 48% for IRSI versus IRSII versus IRSIII groups, respectively, *P* = 0.45). Ten-year PFS rates according to age (±10 year) and primary site (head and neck versus other primary) were statistically different: 62% for patients with H/N DT under the age of 10 versus 32% for patients with no H/N DT and under the age of 10 versus 5% for patients with no H/N DT and over the age of 10 (*P* = 0.01).

## 4. Discussion

This retrospective study with a long followup of a large cohort of pediatric patients with DT confirms the treatment difficulties encountered with this benign tumor, as complete resection is rare, loco-regional progression is frequent and this disease tends to become “chronic,” even in children. The small number of patients and long timescale of this study reflect the rarity of this disease. Treatment modalities obviously change over such a long period of time, resulting in very heterogeneous treatment options that are difficult to compare. Our results support the recently published Italian experience of DT in children [3]. Overall survival is fairly good with a 10-year OS of 88%. Meazza et al. reported a survival of 98.9% based on a fairly similar approach comprising conservative surgery and medical treatment [3].

DT sites were predominantly extra-abdominal in our study, with mainly 42% of limb and 29% of head and neck primary tumors, as reported in other pediatric series [3, 21]. DT of the head and neck (HN) has been reported to occur at a younger age than DT of other sites [11, 12]. In our experience, patients with HN primaries had a better PFS than those with trunk or limb primaries (69% versus 25% versus 14%, *P* = 0.01). Meazza et al. also found a trend toward a better outcome for patients with HN lesions (5-year event-free survival (EFS) of 54.3% versus 33.8% for extremity DT versus 38.5% for trunk sites, *P* = 0.49) [3]. In contrast, other studies have reported a poorer prognosis for HN DT because surgery is rarely complete, with a high risk of relapse, and more aggressive DT adjacent to vital organs [11]. In this study, prognostic factors associated with a good PFS were younger age (<10 years) and HN site. Gender had no impact on PFS. Meazza et al. observed a trend towards a better outcome for males, younger patients, and patients with tumor size <5 cm [3]. Reitamo et al. suggested that the disease is more aggressive in adolescents or more likely to progress in young female adults [22]. In adult series, patients with extremity tumors had a poorer prognosis than those with trunk tumors. Extremity tumors were associated with higher local relapse rates [23]. 

Up until now, surgery has been the mainstay first-line treatment, but complete resection is rare and high recurrence rates are expected. In the present series, surgical margins were histologically free of tumor in only 6% of patients and relapses or local progressions occurred in 61% of the population regardless of the status of the margins. Meazza et al. reported a 5-year EFS less than 45% in this setting. Moreover, some authors have speculated that repeated surgical operations may increase the risk of local progression [11]. Surgery remains difficult at the time of diagnosis of this tumor. Radical mutilating surgery, sometimes performed to achieve complete resection of an invasive form of this relatively benign tumor, should be seriously questioned. Three patients in the present study and 5 out of 94 patients in the Italian series underwent radical mutilating excision only after failure of medical treatment [3]. Mutilating surgery must be considered only after failure of all conservative therapies (several drug regimens, external beam radiotherapy, and even limb salvage with isolated perfusion, when technically possible). 

Marginal resection has been previously reported to be a statistically significant deleterious factor compared to nonsurgical strategies or R0 resection. Some adult or pediatric studies have reported a correlation between the quality of the surgical margins and the risk of recurrence [3]. The IRS grouping was reported by the recent Italian series as the only significant prognostic factor for EFS: the EFS rate was 72% in the IRS I group versus 27% and 35% for the IRS II or III groups, respectively (*P* = 0.0007) [3]. Bonvalot et al. reported similar results on the value of the quality of surgery in a series of 112 adult patients [13]. Due to the small number of IRS I patients in this series, IRS staging was not identified as a prognostic factor in our experience (*P* = 0.45). Some patients may have stable disease for many years without undergoing any treatment [13, 14, 24, 25]. Finally, some recently published data in adults treated by a more conservative policy without front-line surgery or radiation therapy, showed that this can be a safe approach to primary or recurrent DT, which could avoid unnecessary morbidity from surgery and/or radiation therapy [14, 25]. For instance, in an adult population with intra-abdominal tumors, surgery is indicated only when vital organs are involved or when DT is progressive and refractory to drug treatment [13]. Considering the high recurrence rate of DT reported after surgical excision, especially in the case of incomplete resection, first-line surgery at diagnosis should only be considered when large complete tumor resection appears to be feasible without functional or cosmetic damage. In other cases, a strict wait-and-see approach or medical treatment for tumors situated in a life-threatening site may be initiated. The EpSSG NRSTS Committee encourages registration of pediatric patients with DT in the NRSTS 2005 protocol in order to standardize their treatment based on a minimally aggressive strategy comprising a “wait-and-see” strategy designed to avoid repeated resections or destructive surgery and low-dose chemotherapy when systemic therapy is required. At the present time, this wait-and-see strategy is proposed for all pediatric patients with desmoid tumor in a non-life-threatening site and in the absence of marked progression (>30% volume progression). These patients are strictly reviewed every 3 to 4 months, with regular clinical examination and imaging, mostly MRI. 

Adjuvant radiation therapy after surgery appears to improve local control rates compared to surgery alone [12, 24]. In our cohort, five of the nine children who received radiation therapy after initial surgery relapsed (55%). In our opinion, the precise role of radiation therapy as adjuvant therapy has not been clearly established in pediatric patients. High-dose radiation in a growing child can be responsible for growth abnormalities, long-term cosmetic and functional morbidity, and the development of second malignancies. This treatment modality should therefore be used as sparingly as possible in children [12]. Radiation therapy must be considered in life-threatening conditions as the last resort in a growing child and may be proposed as an alternative to mutilating surgery [26]. 

The use of chemotherapy or other systemic agents may be a reasonable alternative to extensive surgery or radiation therapy in a growing child, although some chemotherapy regimens are also associated with a risk of potentially serious adverse effects such as fertility problems, cardiotoxicity, or second malignancies. The efficacy of the various regimens is comparable to that observed in adult soft tissue sarcomas, with a chemo-responsiveness rate of about 40%. Since DT is a slowly growing tumor with a slow response to chemotherapy, prolonged exposure for at least 6 months or even 12-18 months is generally recommended. The low-dose methotrexate and vinblastine combination (MTX-VBL) was used in 19 children of the present series and was associated with a 48% specific response rate, similar to that reported in the few other published studies in adults and children [27]. This low-dose chemotherapy is adequate against this benign tumor due to its definite activity and negligible long-term toxicity with no expected late effects. This treatment is well tolerated by most patients, but requires a central venous catheter and weekly injection with regular blood assessments [27]. Aggressive, rapidly growing, and unresectable DT can be treated by other chemotherapy regimens such as cyclophosphamide, doxorubicin, dacarbazine, or other aggressive regimens [28]. In this case, potential long-term effects should be taken into account. DT is sometimes a slowly growing or mildly symptomatic tumor, in which case, noncytotoxic drugs, such as antiestrogens, NSAIDs, imatinib mesylate, hydroxyurea, interferon-*α*, or retinoic acid can be effective [29, 30]. Hormonal therapy (tamoxifen, toremifene) has also been used in pediatric patients, but with controversial results. These therapies can be combined with chemotherapy with benefit but the long-term clinical improvement is minimal [5] and the late effects in young children are unknown. The experience of treatment in prepubescent children is very limited and this treatment should probably be administered very cautiously. Tamoxifen is a clomifene-like product with an estrogen antagonist activity on mammary receptors and an agonist activity on bone and endometrial receptors. The use of imatinib mesylate for the treatment of DT has provided encouraging results mainly with tumor stabilization [29]. Only 2 patients in the present cohort received imatinib mesylate. Stabilization of disease was obtained in both cases. Balamuth et al. reported a retrospective study of 16 pediatric patients with DT treated with hydroxyurea, but larger studies are needed [31]. 

International prospective cooperation is necessary to develop prospective clinical trials, stratified on clinical and molecular criteria. No biological or molecular data were monitored in our cohort, but CTNNB1 mutations are very common in DT [25, 32]. The intensity of nuclear *β*-catenin expression, p53 expression, or 45F mutations has been reported to be correlated with the risk of recurrence [33]. In the future, molecular analysis could therefore allow selection of patients with a poor outcome and stratification of the treatment of DT patients based on molecular data.

## Figures and Tables

**Table 1:** Clinical characteristics, treatment modalities, and outcome of the 59 patients with desmoid tumors.

Quality of surgical margins	Total (number of patients)	IRS I	IRS II	IRS III		Unknown IRS group
		R0	R1	R2 resection	Biopsy
	*n* = 59	*n* = 3	*n* = 19	*n* = 23	*n* = 12	*n* = 2
*Patients and tumor*						
Gender						
Male	36	2	10	15	7	2
Female	23	1	9	8	5	0
Age at diagnosis (years)						
Median	6.75	5.9	12.8	6.08	9.03	6
Range	0–15	2–14	1–14	0–15	0–15	5–7
Primary						
Head and neck	17	—	4	8	4	1
Wall trunk	9	1	3	4	1	—
Deep trunk/visceral	6	1	1	2	2	—
Limbs	25	1	10	9	4	1
Multiple sites	2	—	1	—	1	—
Predisposing conditions						
Gardner's/FAP	5					
Local surgical trauma	3					
*First-line treatment*						
(i) Initial surgery	47	3	19	23	—	2
No further therapy	35	3	16	16	—	—
Adjuvant therapy:						
RT	9	—	3	4	—	2
CT	3	—	—	3	—	—
(ii) No initial surgery	12	—	—	—	12	—
Neoadjuvant therapy						
Medical treatment then surgery	7	—	—	—	7	—
Medical treatment alone	1	—	—	—	1	—
Wait-and-see strategy	4	—	—	—	4	—
*Outcome*						
Relapse/Progression	38 pts	2 pts	16 pts	15 pts	4 pts	1 pt
5-year PFS	39%	33%	16%	48%		—
Death	7 pts	0	3 pts	3 pts	1 pt	0

Abbreviations: RT: radiation therapy; CT: conventional chemotherapy; PFS: progression-free survival; *n*: number of patients.

**Table 2:** Details on radiation therapy in 21 patients with desmoid tumor.

Disease status at time of RT	Timing of RT	RT dose (Gy)	Long-term status	Site of relapse	Time to relapse or progression after RT (months)	Delayed second RT
Microscopic disease (*n* = 10)	First line	54.4	Relapse	Out	22	—
	First line	50	Relapse	In	12	—
	First line	45	Relapse	In	10	—
	After relapse	45	CRem	—	No
	After relapse	55	CRem	—	No
	After relapse	50	CRem	—	No
	After relapse	50	CRem	—	No
	After relapse	45	CRem	—	No
	Progressive disease	50	CRem	—	No
	Progressive disease	52	Progressive disease	In	20	No

Macroscopic disease (*n* = 12)	First line	50	CRem	—	—
	First line	45	CRem	—	—
	First line	45	CRem	—	—
	First line	50	Relapse	Out	45	—
	After relapse	51	CRem	—	No
	After relapse	50	CRem	—	No
	After relapse	50	CRem	—	Yes
	After relapse	50	Relapse	Out	4	No
	After relapse	45^∗^	Relapse	In	22	Yes
	After relapse	50	Relapse	Out	8	Yes
	After relapse	35^∗^	Progressive disease	In	0	Yes
	Progressive disease	40^∗^	Progressive disease	In	3	No

Negative (*n* = 1)	After relapse	44	Relapse	In	17	No

Unknown (*n* = 2)	First line	45	CRem	—		—
	First line	46	Relapse	Out	0	—

Abbreviations: RT dose: radiation dosage. Microscopic disease: R1 margins after surgery. Macroscopic disease: R2 margins after surgery or no prior surgery (^∗^). Negative disease: surgery with negative margins R0. Unknown disease: status of margins unknown after surgery. Relapse In: relapse in radiation fields. Relapse Out: relapse outside of radiation fields. CRem: complete remission.

**Table 3:** Efficacy of 61 chemotherapy regimens in 32 patients with measurable disease during first or any subsequent line of therapy.

*N*	CR	PR	SD	PD	Unknown response	Overall response (%)	
VA	6	—	**1**	5	—	—	**1 (17%)**
IVA	13	**3**	**1**	9	—	—	**4 (30%) **
VAC	3	—	—	1	1	1	**0 **
VAC and tamoxifen	1	—	—	1	—	—	**0**
Vinblastine-MTX	14	—	**7**	5	1	1	**7 (50%)**
Vinblastine-MTX-tamoxifen	7	—	**3**	3	0	1	**3 (43%)**
Doxorubicin with other agents^∗^	5	—	**2**	0	3	—	**2 (40%)**
Imatinib mesylate	2	—	—	2	—	—	**0**
Others^+^	10	—	**2**	1	7	—	**2 (20%)**

Total	61	**3**	**16**	26 (43%)	14 (23%)	2 (3%)	**19 (31%)**

Abbreviations: VA: vincristine, dactinomycin; IVA: ifosfamide, vincristine, and dactinomycin; VAC: vincristine, dactinomycin, and cyclophosphamide; MTX: methotrexate. *N*: number of patients; CR: complete response; PR: partial response; SD: stable disease; PD: progressive disease.

^∗^Doxorubicin with vincristine, cisplatin, ifosfamide, cyclophosphamide, or tamoxifen.

^+^Others: VINCAEPI (vincristine, carboplatin, and VM 26), MMT95 protocol (IVA, etoposide, epirubicin, and carboplatin), dactinomycin alone, etoposide-ifosfamide, etoposide alone, and carboplatin.
