# Erratum to “Desmoid Fibromatosis in Pediatric Patients: Management Based on a Retrospective Analysis of 59 Patients and a Review of the Literature”

**DOI:** 10.1155/2013/757915

**Published:** 2013-03-06

**Authors:** Caroline Oudot, Daniel Orbach, Véronique Minard-Colin, Jean Michon, Pierre Mary, Christophe Glorion, Sylvie Helfre, Jean-Louis Habrand, Odile Oberlin

**Affiliations:** ^1^Pediatric Oncology Department, Hôpital de la Mère et de l'Enfant, 87042 Limoges, France; ^2^Adolescent and Pediatric Oncology Department, Institut Curie, 75005 Paris, France; ^3^Adolescent and Pediatric Oncology Department, Institut Gustave Roussy, 94800 Villejuif, France; ^4^Pediatric Surgery Department, Hôpital Trousseau, 75012 Paris, France; ^5^Pediatric Surgery Department, Hôpital Necker-Enfants-Malades, 75012 Paris, France; ^6^Radiotherapy Department, Institut Curie, 75005 Paris, France; ^7^Radiotherapy Department, Centre Hospitalier Universitaire de Caen, 14000 Caen, France

We need to update the first and third addresses as shown above. Furthermore, “zero” has been modified and replaced by “—” in Table 3.

## Figures and Tables

**Table 3:** Efficacy of 61 chemotherapy regimens in 32 patients with measurable disease during first or any subsequent line of therapy.

	*N*	CR	PR	Overall response (%)	SD	PD	Unknown response (%)
VA	6	—	1	1 (17%)	5	—	—
IVA	13	3	1	4 (30%)	9	—	—
VAC	3	—	—	—	1	1	1
VAC and tamoxifen	1	—	—	—	1	—	—
Vinblastine-MTX	14	—	7	7 (50%)	5	1	1
Vinblastine-MTX-tamoxifen	7	—	3	3 (43%)	3	—	1
Doxorubicin with other agents*	5	—	2	2 (40%)	—	3	—
Imatinib mesylate	2	—	—	—	2	—	—
Others^+^	10	—	2	2 (20%)	1	7	—

Total	61	3	16	19 (31%)	27 (44%)	12 (20%)	3 (5%)

VA: vincristine and dactinomycin; IVA: ifosfamide, vincristine and dactinomycin; VAC: vincristine, dactinomycin, and cyclophosphamide; MTX: methotrexate. *N*: number of patients; CR: complete response; PR: partial response; SD: stable disease; PD: progressive disease.

*Doxorubicin with vincristine, cisplatin, ifosfamide, cyclophosphamide, or tamoxifen.

^+^Others: VINCAEPI (vincristine, carboplatin, and VM 26), MMT95 protocol (IVA, etoposide, epirubicin, and carboplatin), dactinomycin alone, etoposide-ifosfamide, etoposide alone, and carboplatin.

